# Comprehensive and realistic simulation of tumour genomic sequencing data

**DOI:** 10.1093/narcan/zcad051

**Published:** 2023-09-22

**Authors:** Brian O’Sullivan, Cathal Seoighe

**Affiliations:** School of Mathematical and Statistical Sciences, University of Galway, University Road, Galway H91 TK33, Ireland; School of Mathematical and Statistical Sciences, University of Galway, University Road, Galway H91 TK33, Ireland

## Abstract

Accurate identification of somatic mutations and allele frequencies in cancer has critical research and clinical applications. Several computational tools have been developed for this purpose but, in the absence of comprehensive ‘ground truth’ data, assessing the accuracy of these methods is challenging. We created a computational framework to simulate tumour and matched normal sequencing data for which the source of all loci that contain non-reference bases is known, based on a phased, personalized genome. Unlike existing methods, we account for sampling errors inherent in the sequencing process. Using this framework, we assess accuracy and biases in inferred mutations and their frequencies in an established somatic mutation calling pipeline. We demonstrate bias in existing methods of mutant allele frequency estimation and show, for the first time, the observed mutation frequency spectrum corresponding to a theoretical model of tumour evolution. We highlight the impact of quality filters on detection sensitivity of clinically actionable variants and provide definitive assessment of false positive and false negative mutation calls. Our simulation framework provides an improved means to assess the accuracy of somatic mutation calling pipelines and a detailed picture of the effects of technical parameters and experimental factors on somatic mutation calling in cancer samples.

## Graphical Abstract

**Graphical Abstract:**
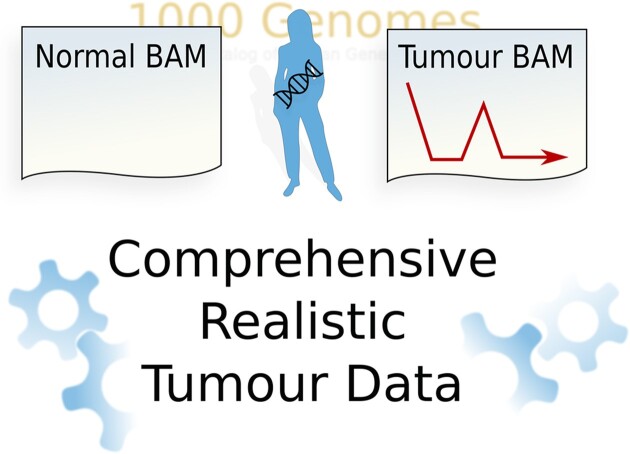

## INTRODUCTION

The identification of somatic mutations from high-throughput sequencing (HTS) data plays a critical role in scientific research and clinical oncology. Cancer driver mutations continue to inform prognosis, guide therapy and shape our understanding of how cancer develops and evolves. Experimental design and analytical decisions, such as sequencing depth (1), target (2) and the choice of bioinformatics pipelines (3–8), all influence the power and accuracy of somatic mutation detection. Assessing their effects on the recovered somatic mutation landscape requires HTS reference data containing a ‘ground truth’ set of somatic mutations for which the location and source of all loci that contain non-reference bases (a base call that does not match the reference genome at that locus) are known. Such data are a key component in the validation and benchmarking of mutation calling pipelines. The accuracy of the results returned by somatic mutation calling pipelines is critical for many research and clinical applications; however, studies benchmarking these pipelines often publish inconsistent results (9–12), indicative of the many challenges faced in this area. The variant allele frequency (VAF) spectrum across all somatic mutations is also relevant for understanding cancer origin and evolution (13) and the development of treatment resistance (14), as well as for inferring clinically important metrics such as tumour mutation burden (TMB), purity and ploidy (15). Significantly, despite the clinical and research relevance of individual mutation frequencies and the VAF spectrum as a whole, no studies to date have attempted to assess the accuracy with which the frequencies of somatic variants are inferred by mutation callers, and this represents a significant knowledge gap.

A number of computational tools have been developed to provide ground truth data to assess the accuracy of somatic mutation callers. Sequencing read simulators, such as ART (16), can be used to generate reads from a reference genome. These are then modified to introduce ‘somatic’ variants (a process known as spiking in variants) using software such as BAMSurgeon (17). Although convenient, a reference-based approach does not reflect the diverse sources of variation within real sequencing data. Increasingly, this is being addressed through a ‘hybrid’ solution employing both real and synthetic sequences (17). Real sequencing data are subsampled into two sets, corresponding to a virtual matched tumour and normal pair, and somatic variants are then spiked into the tumour reads. However, in addition to the variants that are purposely spiked in, these data also contain low-frequency somatic variants present in the source sample from which they were derived, as well as sequencing errors and other artefacts. The precise origin of this additional variation is likely to be unknown, creating difficulties for the evaluation of mutation callers.

The computational tools that are currently used to spike in somatic mutations also have significant limitations. The number of reads that are edited to introduce the mutant allele at the required locus is typically determined by the product of specified mutation frequency and the sequencing depth at the site. This fails to take account of stochastic aspects of the sequencing process. In reality, sequence reads are a random sample of the DNA at a locus and the observed number of reads containing the alternate allele is, therefore, a random variable. Failure to take this into account can result in spike-in bias, where a variant allele is always spiked in if the product of the VAF and the sequencing depth is >1 and never otherwise. For example, a site designated to contain a somatic mutation with a frequency of 10%, which is sequenced to a depth of 30 reads, will always contain exactly 3 reads with the alternate allele if the mutation is spiked in with the widely used tool, BAMSurgeon (17). However, the number of sequence reads containing the alternate allele for a somatic mutation of this frequency may be >3 or <3 in real data.

Here, we describe a comprehensive and stochastic tool for simulating tumour sequencing data that, unlike existing methods, enables precise determination of the accuracy and power to detect a somatic mutation as a function of its actual frequency within the cancer sample. Analysis of simulated data generated using this framework provides novel insights into the relationship between the true somatic mutation frequency spectrum and the empirical frequency spectrum obtained following application of a mutation caller. Using our simulations, we assess mutation caller bias in VAF estimates and demonstrate the empirical somatic mutation frequency distribution corresponding to somatic mutations derived from a neutral model of tumour evolution. We also perform a comprehensive assessment of false positive and false negative somatic mutation calls, made possible by the fact that our simulation tool provides the source of all non-reference alleles in the dataset.

## MATERIALS AND METHODS

**Figure 1.**
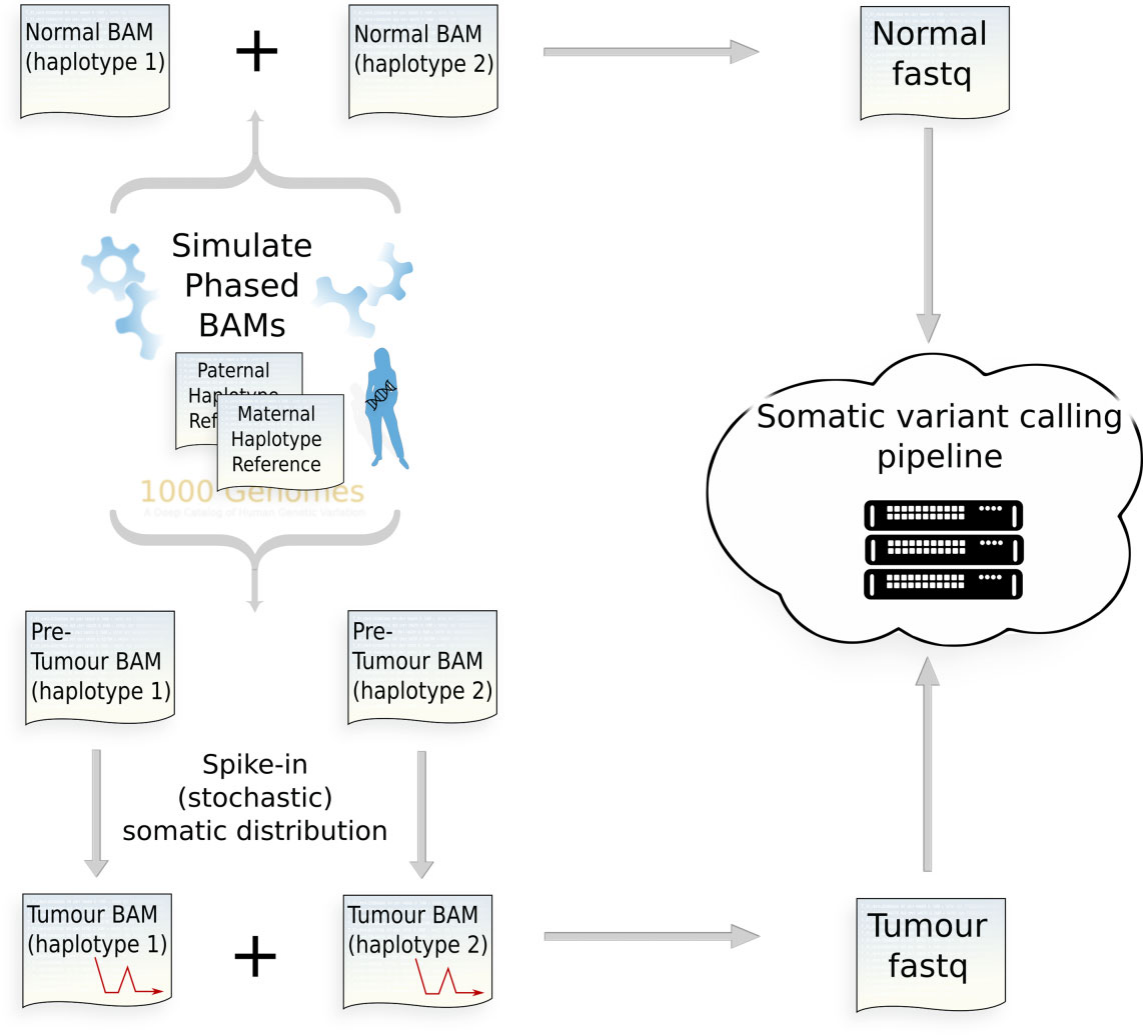
Tumour stochastic HTS simulation framework. Personalized phased donor genome incorporates all single-nucleotide polymorphisms (SNPs) and indels recorded from any 1000 Genomes donor.

A personalized reference genome containing all germline single-nucleotide variants (SNVs) and indels annotated for 1000 Genomes donor HG00110 (female of English and Scottish ancestry) was created. This was used as a base to simulate normal and pre-tumour (i.e. before the somatic variants have been spiked in) genomic sequencing data using the ART read simulator configured with default empirical error profile and corresponding to different depths of coverage, 100×, 200×, 350× and 600×. All reads in the SAM output generated by ART are aligned to their true location within the personalized phased genome from which they were simulated. The target simulated was an exome capture consisting of all hg38 exons plus an additional 100 base pairs at the 3′ and 5′ ends of each capture range. These data were then used as a base to spike in the required somatic distribution of SNVs. Once the spike-in process was completed, the phased data (a BAM pair corresponding to the maternal/paternal haplotype set) were merged and realigned against a standard reference (Figure 1). Realignment was performed using BWA (v0.7.17) (18) with the hg38 reference genome. Somatic variant calling was performed using Mutect2 according to GATK (4.2.2.0) Best Practices for somatic short variant discovery (19). Cross-sample contamination and base quality score recalibration stages were not run as the data were simulated without contamination or systematic biases. Mutect2 was called with argument set to ensure all filtered variants were recorded in the variant call format (VCF) file (tumor-lod-to-emit=0). The stochastic simulation framework is written in C, on the HTSlib 1.13 API (20).

### Simulation of mutation allele frequency spectrum

We used our simulation framework to simulate somatic mutations with defined frequencies. These simulations included the full mutant allele frequency spectrum of a diploid tumour expected under a neutral evolutionary model (13). We also simulated a low-frequency, high-burden point mass at a fixed frequency and finally a uniform distribution of somatic mutation frequencies to investigate caller detection rate and inferred allele frequency as a function of true allele frequency. The first two simulations were repeated across different depths of coverage (100×, 200×, 350× and 600×) to explore the effect of depth on somatic mutation detection and inferred allele frequency. The uniform distribution was simulated at a fixed depth of 100×. Finally, to illustrate the distinction between somatic variant simulation using BAMSurgeon and our simulation framework, the point-mass simulations were repeated using BAMSurgeon to spike in the required burden.

#### Complete allele spectrum simulation of a diploid tumour derived from a neutral evolutionary model

**Figure 2.**
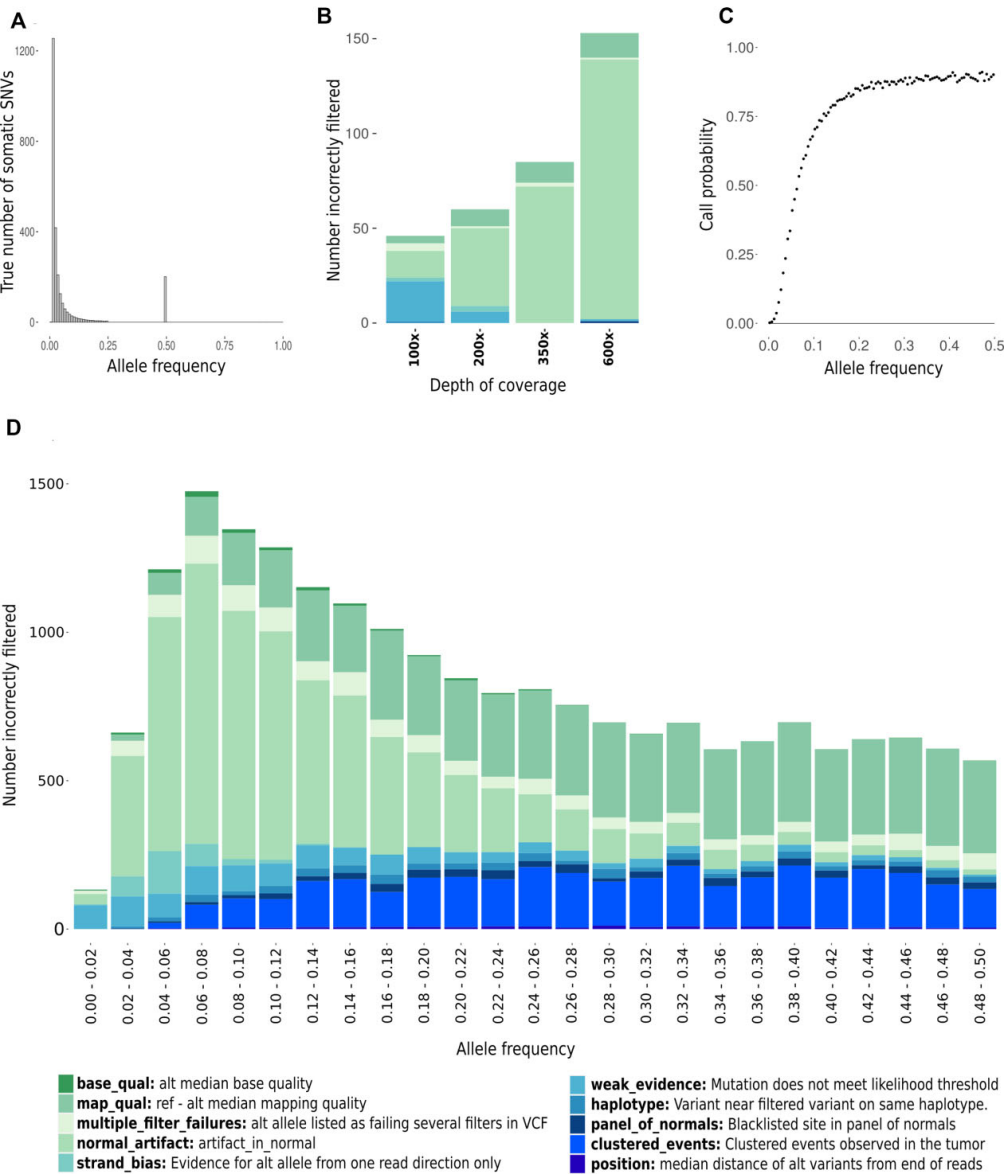
(**A**) The ‘ground truth’ or true frequency spectrum of somatic mutations in our simulation of a diploid tumour derived from a neutral evolutionary model. The total true burden was 2681 somatic variants. (**B**) Number of true somatic variants incorrectly filtered by Mutect2 as artefacts, stacked by filter type, from neutral model simulations at each of the four sequencing depths, 100×, 200×, 350× and 600×. (**C**) Probability that a true somatic mutation is passed by Mutect2 as a function of its true allele frequency for 100× sequencing data with 100% tumour purity. This simulation was also repeated over a range of depths on a reduced target size (Supplementary Figure S1). (**D**) Number of true somatic variants incorrectly filtered as artefacts in the uniform frequency simulations, stacked by filter type. Each bar represents the fractions of false negatives incorrectly excluded by the caller from a total somatic burden of 40 000 variants.

A total burden of 2681 somatic variants within a true frequency range of 0.01–0.25 was simulated, with a VAF spectrum for subclonal mutations corresponding to the neutral model of evolution (13) (Figure 2A) (i.e. cumulative distribution function of the mutation frequency, *f*, proportional to 1/*f*). The clonal mutations were simulated with a fixed frequency of 0.5 (the simulation had 100% tumour purity). Mutations were spiked into each of the four pre-tumour phased BAM pairs (with depths of 100×, 200×, 350× and 600×). Mutect2 was then run on the resulting matched tumour–normal pairs. The resulting variant output (VCF) was compared to the ground truth values.

#### Low-frequency, high-burden point-mass somatic distribution

A low-frequency burden of 10 000 somatic variants, all with a true allele frequency of 0.035, was spiked into each of the four pre-tumour phased BAM pairs (100×, 200×, 350× and 600×) using the stochastic simulation framework. Mutect2 was then run on the matched tumour–normal pairs. The resulting variant output (VCF) was correlated against its ground truth values (again using the simulation framework). The observed VAF spectrum was plotted showing the dispersion of allele frequencies about their ground truth at each of the four sequencing depths.

#### Uniform somatic distribution

We divided the first half of the allele frequency range (i.e. frequencies from 0 to 0.5) into 100 semi-centile bins. Into each bin, we spiked in a uniform distribution of 10 000 somatic variants at loci randomly distributed across the target region in 100× pre-tumour HTS data. For each semi-centile, we recorded the percentage of the ground truth burden that was passed (considered true somatic) or filtered (considered artefactual) and its associated allele frequencies, as inferred by Mutect2. From this, we created a matrix detailing the detection rate for each semi-centile and the regions of the spectrum in which that burden was detected, as annotated by Mutect2. This enables us to predict how the caller performs in detecting a true burden and identifying its associated allele frequencies. The reasons candidate somatic variants were filtered by the caller in each semi-centile were also recorded. The simulations were carried out in groups of four semi-centiles per simulation, with each simulation containing a total burden of 40 000 somatic variants, yielding 25, 100×, tumour–normal pairs that were subsequently analysed with Mutect2.

#### Simulations of FFPE and 8-oxoG artefacts

We performed additional simulations that included artefacts that are typical of formalin-fixed, paraffin-embedded (FFPE) samples and oxidative DNA damage. To simulate artefacts associated with FFPE samples, we downloaded high-coverage (minimum depth ≥500) colorectal variant call data (21) from three patients, each containing two samples, one fresh frozen (FF) and the other FFPE (48 h fixation time), both of which had been resected from the same tumour. Using variants identified only in the FFPE sample, we estimated the FFPE single-base substitution (SBS) signature (based on the conventional 96 triplet mutation types) associated with these data and created a context-specific FFPE distribution of simulated artefacts. We then created a second distribution based on COSMIC signature SBS45 (22) to simulate oxidative damage during sample preparation. Both distributions were combined with an empirical set of DNA damage artefact allele frequencies (21). To preserve the required orientation, the 100× pre-tumour BAMs were each split into two separate files, one with reads from inserts that aligned to the forward genomic strand and the other containing the remaining reads. The target burden, totalling 7 332 528 DNA damage artefacts, was then divided evenly between forward and reverse strand alignment BAM files and spiked in using the stochastic simulation framework. All files were merged back into the final tumour BAM on completion and realigned against the hg38 reference before being subjected to variant calling, using Mutect2 in tumour–normal mode. These simulations did not include any somatic mutations and, consequently, any variants identified by the caller were false positives.

## RESULTS

The simulation framework developed here, which we refer to as stochasticSim, has significantly enhanced functionality compared to existing methods (Table 1). A key feature is the fully comprehensive truth set. Truth sets based on data derived from controlled mixtures of distinct samples (usually cell lines) or by spiking in mutations into a single sample contain germline variants, sequencing errors and alignment errors, among other artefacts. They also contain true somatic mutations present, usually at low frequencies, in the original samples. As a consequence, these simulation methods do not provide an accurate and complete set of the true somatic mutations in the sample (24,25). This is required, for example, for an accurate assessment of the performance of methods to detect somatic mutations. In contrast, a comprehensive truth set not only allows us to identify true and false positives definitively, but also enables us to identify the cause of all false positive calls.

**Table 1.** Comparison of the functionality of tumour simulation methods

	Features												
	Germline simulation			Somatic simulation			Alignment						
Method	SV/CNV	SNP	Indel	SV/CNV	SBS	Indel	Pre-spike-in	Post-spike-in	Haplotype aware	Context aware	Orientation aware	Stochastic	Comprehensive truth set
Stochastic simulation	No	Yes	Yes	No	Yes	No	True	hg38	Yes	Yes	Yes	Yes	Yes
BAMSurgeon 2018 (17)	No	No	No	Yes	Yes	Yes	Estimate	hg19	Yes	No	No	No	No
Cell line admixture (23)	No	No	No	No	Yes	No	Estimate	hg19	No	No	No	Yes	No

Somatic indel simulation is not yet supported by stochasticSim. All germline indels, which account for the vast majority of indels in tumour samples, are simulated, however. CNV, copy number variation; SBS, single-base substitution; SNP, single-nucleotide polymorphism; SV, structural variation.

Our simulation framework provides a complete record of the source of all non-reference bases in the data. This allows us to assess the sources of all false positive and false negative mutation calls. In the first simulation, a total of 2681 somatic mutations were simulated across a range of frequencies corresponding to a neutral model of tumour evolution (13) (Figure 2A), with sequencing depths ranging from 100× to 600×. The false positive rate was extremely low, with just one false positive (due to sequencing error) being detected over four simulations at different sequencing depths. Overall detection rates at each of the four sequencing depths (100×, 200×, 350× and 600×) were 22%, 28%, 32% and 36%, respectively. The relatively low proportion of mutations detected in this simulation reflects the large proportion of low-frequency variants implied by the neutral model of tumour evolution (∼75% of the mutations had a true allele frequency <0.05). As the number of reads carrying the alternative allele is a random variable, a number of mutant alleles were not found at all in the simulated data and therefore would not be detected by any mutation calling pipeline. At each of the four sequencing depths (100×, 200×, 350× and 600×), 24%, 13%, 8% and 4%, respectively, of the true somatic burden received no coverage of the alternative allele at the variant locus. The majority of the remaining true somatic variants were present at too low frequencies to be considered by Mutect2 for filtering and were dropped without leaving any record in the VCF file (Figure 2).

A small proportion of all somatic variants (∼2% at 600×), most of which were also of low allelic frequency, were missed as the reads supporting the alternative allele were incorrectly aligned against the reference genome. A substantial number of the true somatic mutations were removed by mutation caller filters that incorrectly identified them as artefacts (Figure 2B). The contribution of different filters varied across sequencing depths. Interestingly, the number of true somatic mutations that failed these filters increased with increasing sequencing depth. This was mainly due to the alternative allele being incorrectly flagged as a germline or other artefact common to both the tumour and normal samples (normal_artifact). As sequencing depth increases, so too does the probability of a read error in the normal sample occurring at the same locus and with the same base as a true somatic variant in the tumour, thereby increasing the number of variants filtered in this way.

### Probability of somatic mutation detection as a function of mutation frequency

To investigate the relationship between the probability of detecting a somatic mutation and its frequency in the tumour sample, we simulated somatic mutations distributed uniformly over a range of frequencies from 0 to 0.5. The true number of somatic variants together with the total number detected by the caller in each semi-centile was recorded, allowing us to assess the sensitivity to detect somatic variants over a range of allele frequencies. As expected, the detection rate (defined as the probability of a true somatic mutation being passed by the caller) was a strong function of the simulated variant frequency (Figure 2C). The normal_artifact filter accounted for ∼34% of true somatic variants incorrectly filtered by Mutect2 with a significant contribution at lower frequencies (<0.2) (Figure 2). The number of true somatic mutations removed by the clustered_events filter increased with increasing frequency (Figure 2D). This number was relatively high in these simulations due to the high burden of mutations simulated in each frequency band (40 000 somatic variants, randomly distributed across a 76 Mb target region). This high burden increased the probability of multiple somatic mutations being spiked in in close proximity, resulting in them being flagged by this filter. A small number of true somatic mutations were filtered due to strand bias, but this was not noticeable beyond an allele frequency of 0.1 and it should be noted that no strand-specific artefacts were simulated.

### Empirical mutation frequency spectrum corresponding to a neutral model of tumour evolution

**Figure 3.**
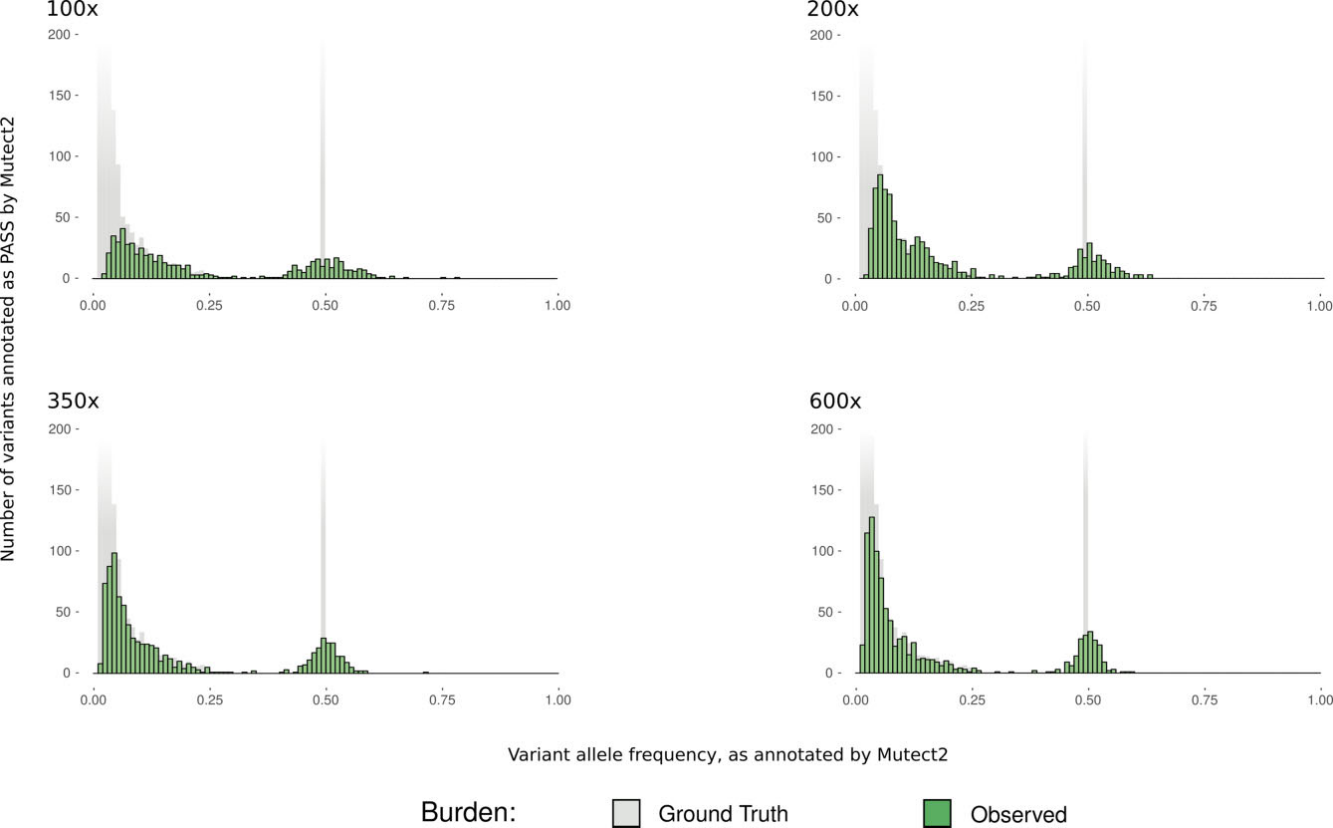
VAF plots from Mutect2 output of a diploid tumour derived from a neutral evolutionary model, overlaid on the ground truth. Ground truth burden is faded where it starts to extend beyond the *y*-axis. Depth of coverage is as indicated in each plot.

Information on how tumours evolve is relevant for gaining a better understanding of cancer origin, the development of immune evasion and resistance to treatment. Models of tumour evolution have implications for the frequency spectrum of somatic mutations observed in a cancer sample; however, the relationship between a theoretical frequency spectrum and the empirical spectrum that is observed when mutations are called using existing computational pipelines is unclear, particularly in the case of moderate sequencing depth. We assessed the mutation frequency spectrum recovered by the caller for the simulations corresponding to the neutral model of evolution (13) over a range of sequencing depths (100×, 200×, 350× and 600×). These empirical distributions differ qualitatively for different sequencing depths, with lower depth simulations having a much higher proportion of mutations at intermediate frequencies than predicted by the neutral model of tumour evolution (Figure 3). As expected, the observed frequency spectrum resembled more closely the expected form (with a cumulative distribution function proportional to the reciprocal of the frequency) at the higher sequencing depths. The inferred frequencies of somatic mutations are also relevant for the calculation of TMB, which is typically defined as the number of somatic SNVs per megabase (mut/Mb) with an inferred frequency ≥0.05. The ground truth TMB for the diploid tumour in this simulation was 8.5 mut/Mb (equivalent to 645 variants with a true frequency ≥0.05, at 100% tumour purity for a 76 Mb target). TMB was estimated at each of the four sequencing depths (100×, 200×, 350× and 600×) as 6.99, 7.70, 7.84 and 7.84 mut/Mb, respectively, with a 12% increase in estimated TMB between 100× and 600×.

### Misestimation of mutation frequencies

**Figure 4.**
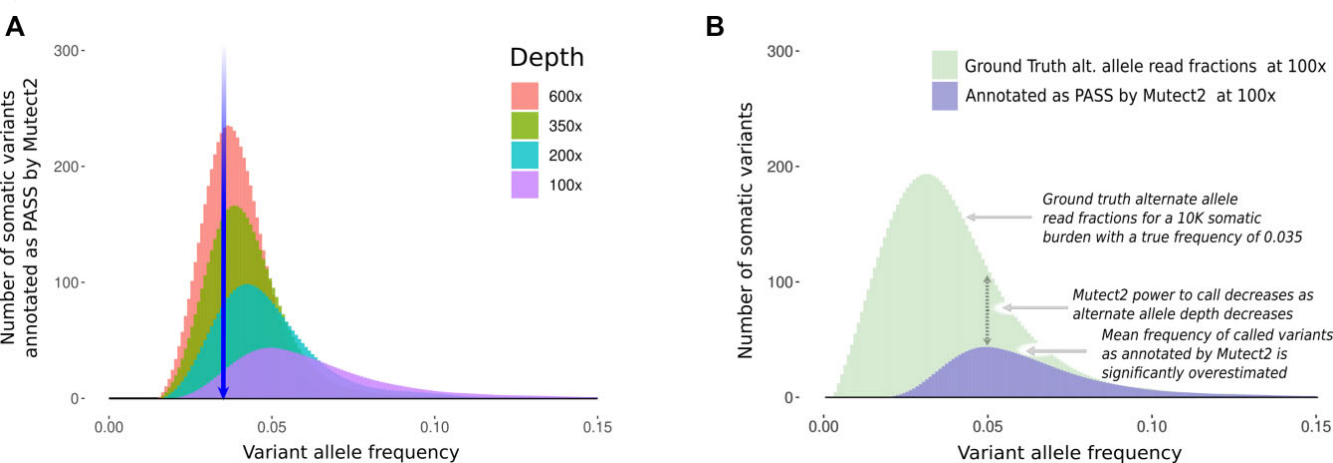
(**A**) The VAF distribution as inferred by Mutect2 from simulated data consisting of 10 000 somatic mutations, each with a true allele frequency of 0.035. The blue arrow indicates the true allele frequency at which the somatic burden is located (the ground truth). Each of the overlay plots indicates what is inferred by Mutect2 at the sequencing depths indicated. The data have been processed using the smooth.spline function from the base R stats package (26). The same data are also available without smoothing (Supplementary Figure S4). (**B**) Explanation of the somatic variant low-frequency caller bias, as annotated by Mutect2 for the 100× data from the previous panel.

To illustrate the impact of stochastic effects on the estimation of somatic mutation frequencies, we simulated 10 000 somatic mutations at a fixed frequency of 0.035. The detection rate (i.e. percentage of the somatic mutations annotated as PASS by Mutect2) at sequencing depths of 100×, 200×, 350× and 600× was 20%, 33%, 45% and 54%, respectively, with very small numbers of false positive somatic mutations at each depth (6, 2, 1 and 3, respectively, with one of the false positives resulting from incorrect read alignment and the remainder from sequencing error). The mean inferred frequencies returned by the caller were 0.065, 0.050, 0.044 and 0.040, illustrating an upward bias (relative to the true frequency of 0.035; Figure 4A), which decreases with increasing sequencing depth. The bias results from the relationship between the probability of detecting a somatic mutation and the number of reads containing the mutation. As we move to the right of the spectrum (Figure 4B), the fraction of the variants recovered (ratio of the height of the pass to ground truth histograms) increases. This results in an allele frequency distribution for pass variants with a mode that is shifted to the right, relative to the ground truth distribution. We have identified similar biases in VAFs previously, in the context of the use of a mutation frequency threshold in the calculation of TMB (27). However, as seen in these simulations, a threshold is not required to observe a bias in the inferred variant frequencies (which are estimated from the read fractions). We also used these simulations to compare our stochastic simulation toolkit with read fraction methods of simulating HTS data by repeating the simulation using BAMSurgeon (17) to spike in the required distribution. A similar total burden was detected by Mutect2 from both simulations (BAMSurgeon totals: 2130, 3016, 4376 and 5303; stochastic simulation totals: 2008, 3349, 4473 and 5398); however, there were substantial differences in the VAF spectrum associated between the two cases (Supplementary Figure S2).

### Simulations of FFPE and 8-oxoG artefacts

FFPE is a method of tissue preservation enabling samples to be stored at room temperature almost indefinitely (28). The procedure also creates asymmetric DNA damage such as deamination of cytosine to uracil (resulting in the detection of C>T transitions) (21). Similarly, oxidative DNA damage introduced during sample preparation, for example, as a by-product of acoustic shearing (29), generates 8-oxoguanine (8-oxoG) leading to G>T transversions. Both types of DNA damage usually manifest as a high burden of low-frequency artefacts in sequencer output. Only a small proportion of the simulated FFPE and 8-oxoG burdens (11 283 DNA damage artefacts from a true total of 7 332 528, or 0.0015) met the required (GATK) significance threshold to be considered for filtering. Either the remainder was ignored by Mutect2 or no reads carrying the artefacts were recovered from the simulation output. Of these, 7072 were correctly removed by standard Mutect2 filters (such as weak_evidence or base_qual). An additional optional filter in Mutect2, which checks for evidence of orientation bias in the variant call, removed most (99%) of the artefacts that made it through the standard set of filters. However, even after this optional filter, 42 variants corresponding to simulated FFPE and 8-oxoG artefacts were incorrectly annotated as PASS. Although the 42 artefacts that made it through all filters represented only a very small proportion of the original number of sites at which artefacts were simulated, this number could have a substantial impact on results in many studies. A large-scale empirical analysis of TMB in over 100 tumour types indicated a median value of 2.7 mut/Mb (30). This would translate as 205 somatic mutations on the simulation target used in this study. Together with 42 artefacts incorrectly annotated as PASS, this would imply a false positive rate of 20%.

As expected, the mutational profile detected by Mutect2 resembles an FFPE and 8-oxoG DNA damage signature (Supplementary Figure S3). Interestingly, the total median depth at true negative loci (where the DNA damage artefact was correctly filtered by Mutect2) was 95, as opposed to 50 at false positive loci (where the damage was incorrectly passed). The median number of reads supporting the alternative allele recorded by the caller was 5 at true negative loci and 3 at false positive loci, with median alternative allele frequencies of 0.054 and 0.084, respectively. In the case of true negatives, 4043 out of a total of 4169 true negatives contained evidence supporting the candidate somatic allele on one genomic strand only. The remaining records contained evidence from both genomic strands with the evidence from one strand caused by sequence error. In the case of false positives, however, only 30 out of 42 recorded evidence of the alternate allele from one genomic strand only, suggesting interaction between false positives arising from DNA damage and the occurrence of the same substitution due to sequencing error on the opposite strand (*P* = 5e−09, from Fisher’s exact test). In effect, in the case of 12 false positives, a sequence error enabled the DNA damage artefact to escape the orientation bias filter as evidence of the alternative allele was present on both genomic strands. To explore the scenario in which histologically normal tissue adjacent to the FFPE tumour sample is used as a control, we spiked in the same level of FFPE and 8-oxoG burden to the normal sample and re-ran the somatic variant calling pipeline. This simulation yielded similar results (34 false positives with 12 showing evidence of the alternate allele on both strands).

## DISCUSSION

We have developed a computational framework for simulating personalized, phased, cancer genome sequencing data that creates a somatic SBS cancer distribution in a base BAM file containing all germline indel and SNV variation from a 1000 Genomes donor. Cancer indel and structural variants are not yet simulated by this framework. Our framework provides a comprehensive report on the sources of all non-reference sites in the simulated data and accounts for the randomness in the number of reads that contain the non-reference allele at somatic mutation sites. We have applied this framework to assess the performance of a widely used pipeline to call somatic mutations. In agreement with previous reports (3,9,31), our initial analysis indicated that the GATK4 Mutect2 pipeline had a very low rate of false positive mutation calls. However, pre-analytical factors, particularly those associated with sample storage and preparation, can significantly impact downstream somatic variant analysis. Artefacts introduced in these stages are overlooked by current bioinformatics simulation methods (Table 1) and not accounted for in their assessment of caller specificity. To illustrate this, we tested the GATK orientation bias filter against simulated FFPE and 8-oxoG damaged sequencing data and demonstrated a mechanism by which orientation bias artefacts escape GATK filtering leading to additional caller false positives. We also quantified the number of false negatives, corresponding to true somatic variants that were incorrectly filtered by Mutect2, and investigated biases in allele frequency estimation.

Misestimation of allele frequency may be of particular scientific and clinical relevance. We have previously reported a bias in the inferred mutation frequency when only mutations with an observed frequency greater than a threshold are considered (27). Despite the absence of an explicit threshold, simulations in this paper reveal a similar allele frequency bias resulting from the dependence of mutation detection probability on the number of reads that support the mutant allele (Figure 4). The mutation frequencies at which this bias is observed decrease with increasing depth of coverage (Figure 4). The novel simulation framework enabled us to investigate such biases in realistic simulated data using a commonly applied somatic mutation calling pipeline. It also allowed us, for the first time, to determine the observed frequency spectrum that results when mutations from a theoretical spectrum corresponding to a model of tumour evolution are called from cancer sequencing data.

We found that a substantial number of true somatic variants were excluded by the caller as a consequence of being incorrectly identified as an artefact common to both tumour and normal samples. Mutect2 filters candidate variants based on minimal evidence of their presence in the normal sample (normal_artifact), even when the variant is present at much greater frequency in the cancer sample. This means, along with filtering a number of (primarily germline) true negatives, true somatic variants can also be removed due, for example, to sequencing errors in the normal sample. Our simulations, which used the same depth of coverage in both tumour and normal samples, demonstrated that this can be a significant issue, particularly at high depths. For example, one variant was incorrectly flagged as normal_artifact based on its detection by Mutect2 at an allele frequency of 0.00054 in the 600× normal sample. The allele in the normal sample was, in fact, a sequencing error. The median allele frequencies in the normal sample for which normal_artifact false negatives were excluded at depths 100×, 200×, 350× and 600× were 0.0064, 0.0039, 0.0022 and 0.0016, respectively. In practice, the normal sample is often sequenced to a lower depth than the cancer sample. This would reduce the number of mutations that are lost in this way. However, some assays require the same depth of coverage in the normal sample (e.g. copy number analysis), while another publication recommends as high a depth of coverage as possible in both tumour and normal samples (32). We recommend manual curation of variants filtered solely as normal_artifact, particularly where they may be of clinical relevance.

## CONCLUSION

High-confidence identification of somatic mutations in tumour samples and accurate inference of their frequencies are important for clinical decision-making and in cancer research. Realistic simulations continue to play a key role in this regard, improving our understanding of the performance of computational pipelines that have been designed to identify somatic mutations. The extremely low false positive rates achieved by somatic variant callers such as Mutect2 (3,9,31) are in part enabled by an extensive set of filtering steps designed to remove artefacts. However, we have demonstrated that strict thresholds enforced by some of these filters come at a price, in terms of power, with some true mutations being flagged by the filters. We have highlighted limitations in existing methods of assessing the false positive rates of mutation callers and demonstrated a mechanism through which DNA damage introduced during the pre-analytical phase of the sequencing process can lead to false somatic mutation calls. We have also quantified the extent of the bias in the estimated frequencies of the somatic mutations that are identified, as a function of sequencing depth, and determined the empirical mutant frequency spectrum corresponding to the neutral model of tumour evolution. Our simulations also allow us to predict caller detection rate as a function of allele frequency. This novel simulation tool can be applied to evaluate the accuracy with which individual mutations or mutation burdens are calculated and to compare the observed frequencies of somatic mutations to their expected distribution under competing models of tumour evolution.

## Supplementary Material

zcad051_Supplemental_Files

## Data Availability

The software and results relating to this publication are available at Zenodo with doi:10.5281/zenodo.8155004. The stochastic simulation framework is also available from https://github.com/BrianOSullivanGit/stochasticSim.
