# Supplementary material for: Comprehensive and realistic simulation of tumour genomic sequencing data
Source: NAR Cancer. 2023 Sep 22;5(3):zcad051. doi: 10.1093/narcan/zcad051 (PMC10516706; doi:10.1093/narcan/zcad051)
Supplement: zcad051_Supplemental_Files [file zcad051_supplemental_files.zip › suppinfo_update7.pdf]

*Published online DD MM YYYY* *Nucleic Acids Research, YYYY, Vol. xx, No. xx* **1–4**
*doi:10.1093/nar/gkn000*

# SUPPLEMENTARY INFORMATION: Comprehensive and realistic simulation of tumour genomic sequencing data.

**CHROMOSOME 19, SENSITIVITY VS TRUE ALLELE FREQUENCY AT 50X, 200X AND 600X**

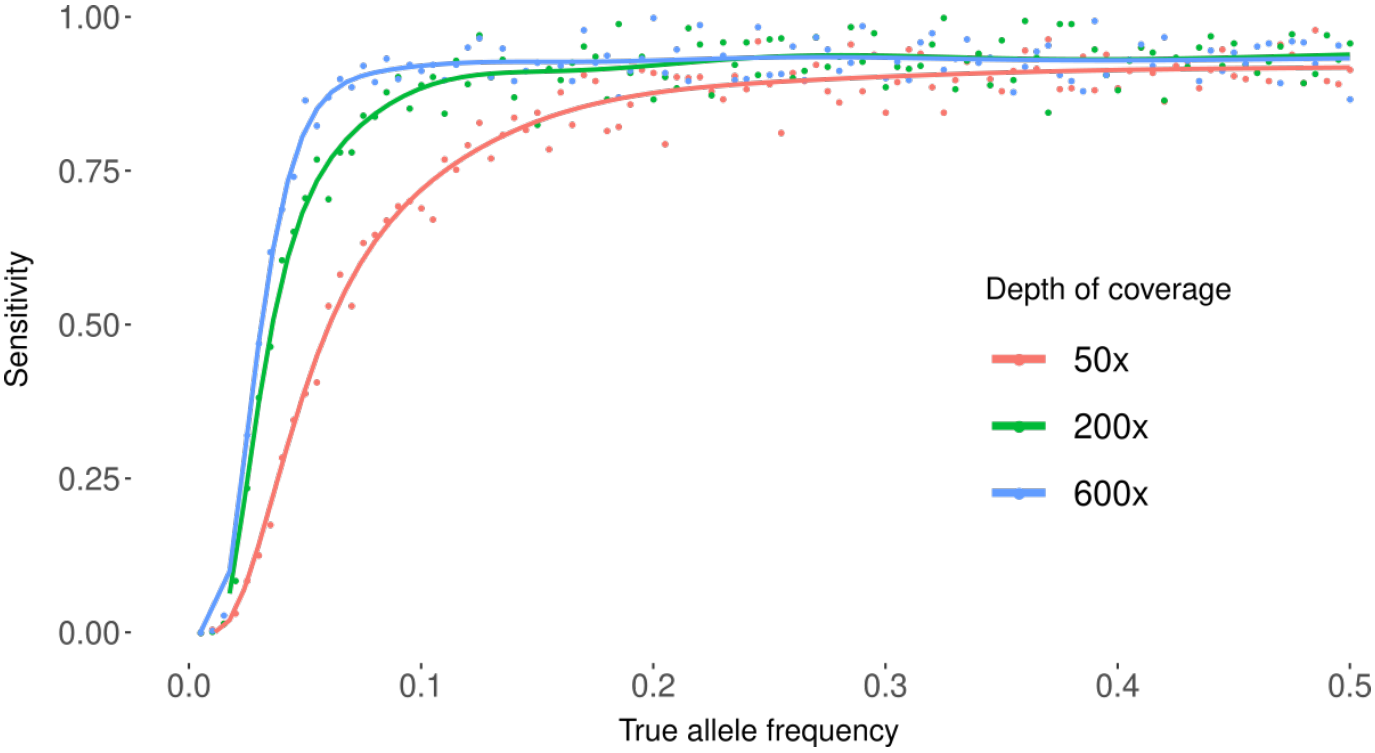


**Supplementary Figure 1.** A comparison of sensitivity vs true allele frequency on chromosome 19 across three depths of coverage. Plots are valid for a chromosome 19 exome target only.

© YYYY The Author(s)
This is an Open Access article distributed under the terms of the Creative Commons Attribution Non-Commercial License (http://creativecommons.org/licenses/by-nc/2.0/uk/) which permits unrestricted non-commercial use, distribution, and reproduction in any medium, provided the original work is properly cited.

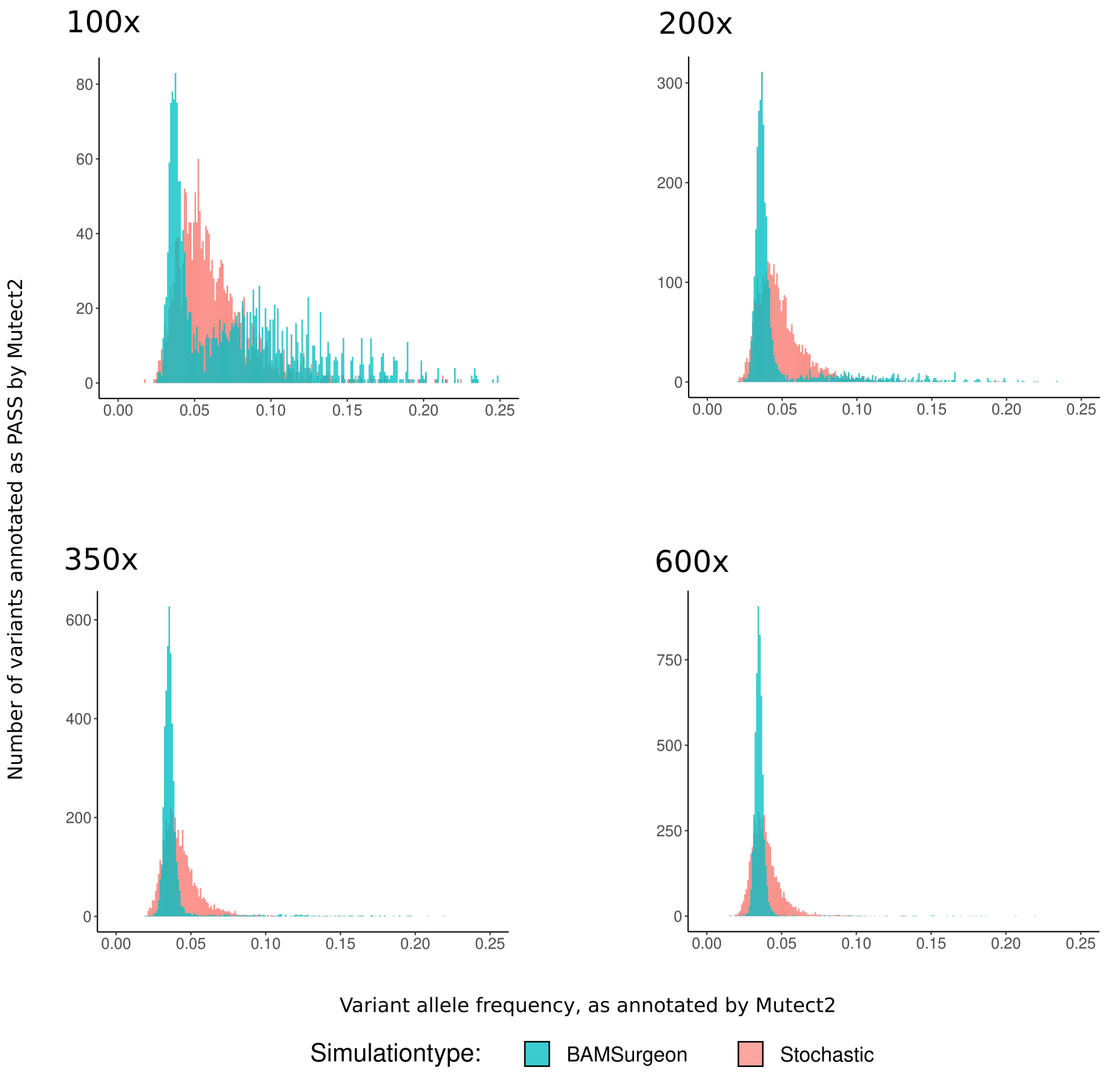


**Supplementary Figure 2.** The VAF distribution as inferred by Mutect2 from separate sets of data simulated using competing stochastic and read fraction (BAMSurgeon) methods over depths of coverage 100x, 200x, 350x and 600x. The ground truth, a clonal point mass of 10k somatic variants centred at a true frequency of 0.035 in simulated genomic sequencing data from a personalised diploid genome, is the same for all simulations. Plots are overlaid by the spike-in method (read fraction versus stochastic).

## SPECTRAL COMPARISON OF BAMSURGEON VERSUS STOCHASTIC SIMULATION

In read fraction simulation methods, the number of reads at the pileup designated for spike-in is chosen to ensure the resulting alternate allele read fraction and true variant frequency match. To illustrate the advantages of our stochastic simulation methods we repeated the point mass simulations using BAMSurgeon, a widely used read fraction method for creating tumour genomic sequencing data. BAMSurgeon simulations were characterised by a sharp peak around the target frequency that does not reflect the distribution of allele frequencies observed from actual NGS data. By default, BAMSurgeon will also 'force' the insertion of the alternate allele into a read if its thresholds relating to the minimum number of reads containing the alternate allele are not met. This artificially inflates the observed allele frequency at a significant number of spike-in loci. It is most noticeable at lower depths, as can be observed at 100x and to a lesser extent, 200x, resulting in a significant portion of the burden lying to the right of the spike (Figure 4). In contrast, the stochastic approach incorporates additional variation introduced by random changes in the proportion of alternate alleles picked up at each target locus during the sequencing process, giving a wider dispersion of observed allele frequency at each sequencing depth and a more realistic simulation of the mutation frequency spectrum (Figure 2). With stochastic simulation, as with actual genomic sequencing data, the presence of the alternate allele in a read may also be masked by sequencing error should it occur.

## PREANALYTICAL DNA DAMAGE PROFILES

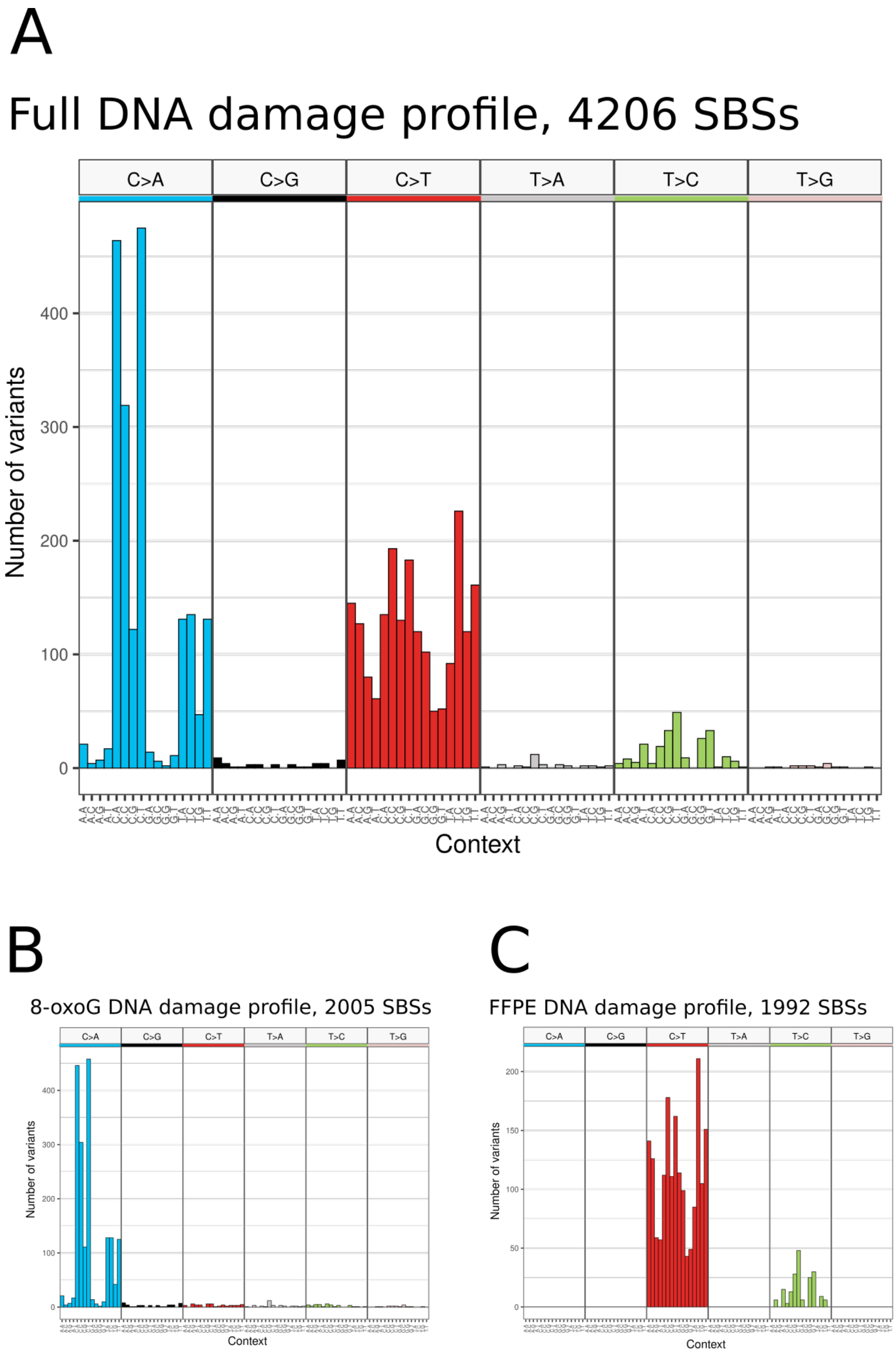


**Supplementary Figure 3.** TNC profile of simulated DNA damage introduced to the tumour sample during preanalytical stage of sequencing as detected by Mutect2.**A:** Complete profile, containing all damage within the sample. **B:** Damage exclusively caused by oxidation. **C:** Damage exclusively caused by FFPE processing.

## APPLICATION OF DATA SMOOTHING TO POINT MASS CALLER OUTPUT

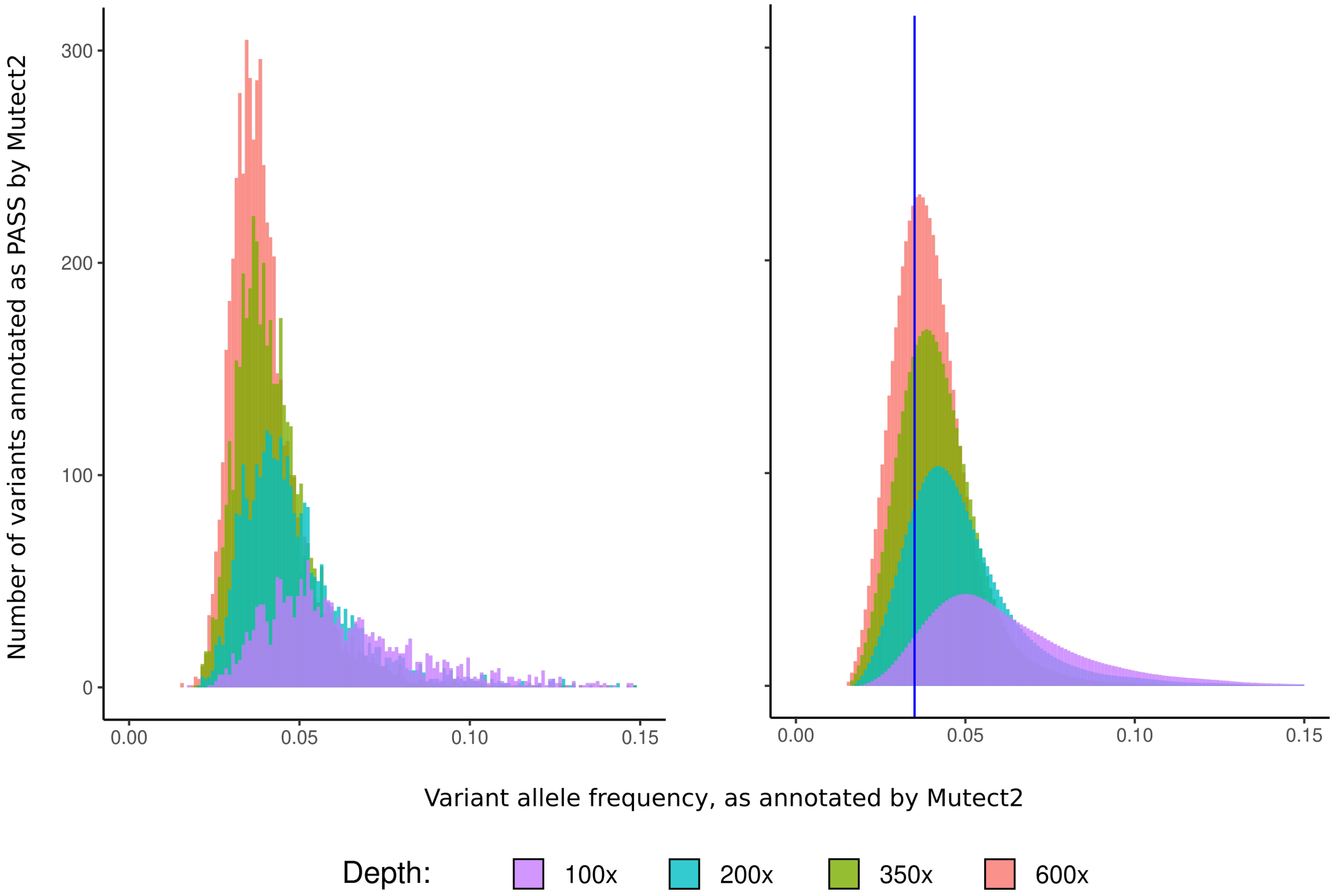


**Supplementary Figure 4.** Empirical VAF distribution as inferred by Mutect2 from simulated data consisting of 10,000 somatic single nucleotide variants each with a true allele frequency of 0.035 at the sequencing depth indicated. The blue line indicates the true allele frequency at which the somatic burden is located. The plot on the left shows what is inferred by Mutect2 at each depth of coverage. The same plot is shown again on the right after the data has been processed using the smooth.spline function from the base R stats package.

We used data smoothing to help illustrate how decreasing depth of coverage attenuates Mutect2's power to call at progressively increasing somatic allele read fractions. Somatic variant callers rarely call a variant based on the evidence of a small number of reads containing the alternative allele. This causes a bias (Figure 4), in caller allele frequency estimation, the extent of which depends on depth of coverage. As depth of coverage increases the distribution mode starts to approach the true allele frequency at which the somatic burden is located and the bias in the allele frequency estimated by the caller decreases. The frequency distribution plotted without data smoothing is also included for reference (Figure 4).
