# Supplementary figures and images for: Comprehensive and realistic simulation of tumour genomic sequencing data
Source: NAR Cancer. 2023 Sep 22;5(3):zcad051. doi: 10.1093/narcan/zcad051 (PMC10516706; doi:10.1093/narcan/zcad051)

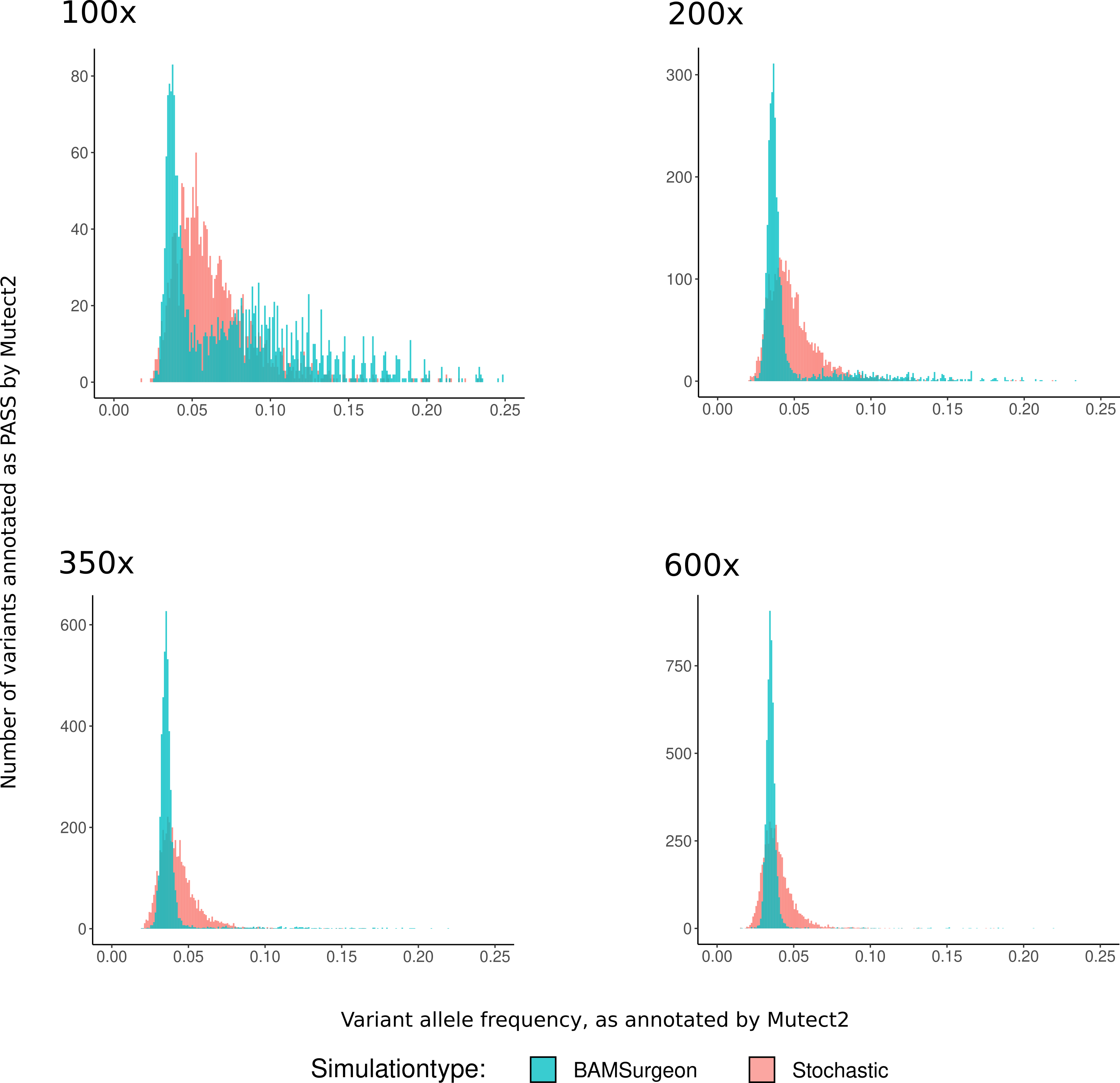

Supplement: zcad051_Supplemental_Files [file zcad051_supplemental_files.zip › bsVsSto.png]

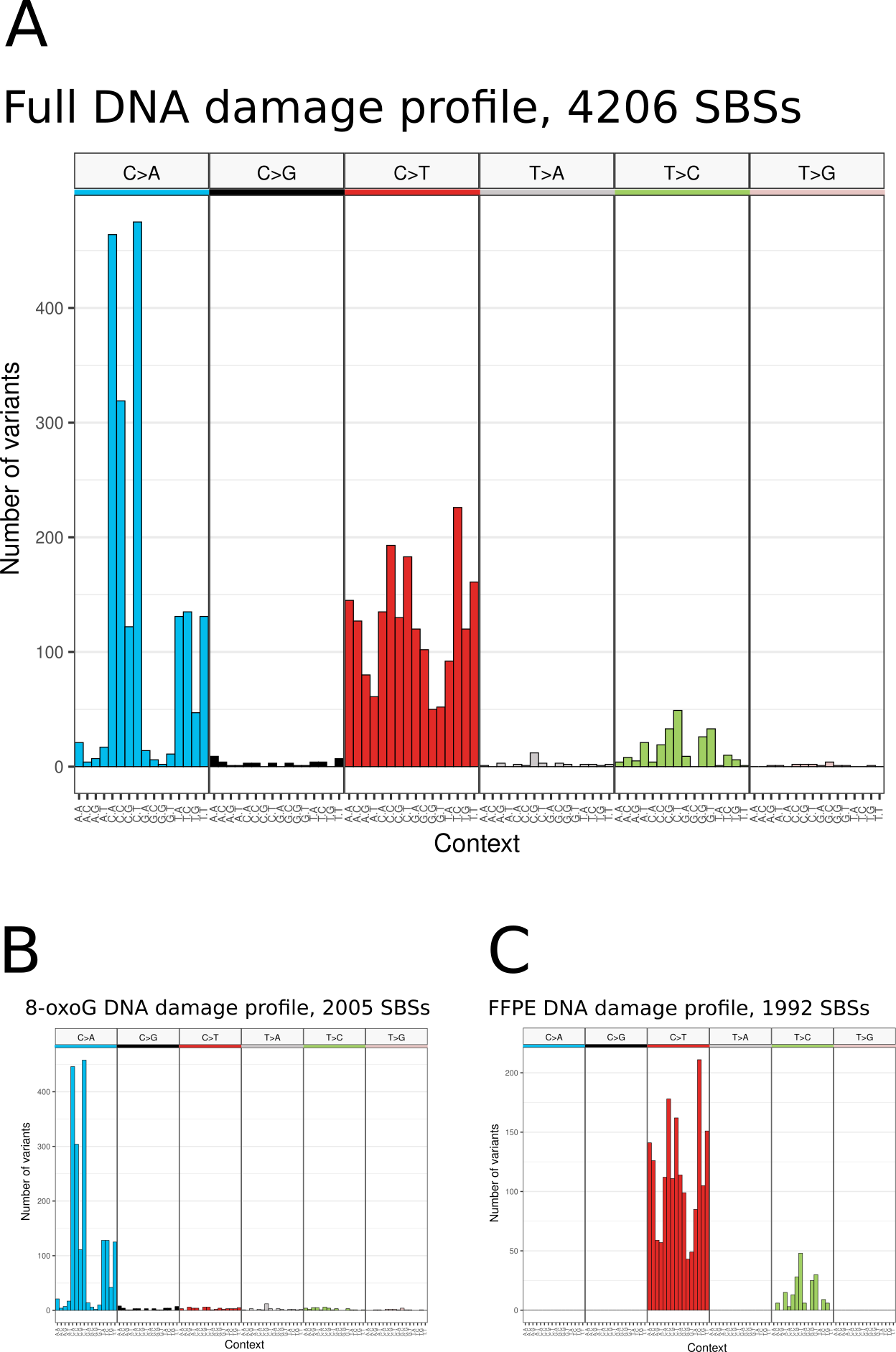

Supplement: zcad051_Supplemental_Files [file zcad051_supplemental_files.zip › DNAdamageProfile.png]

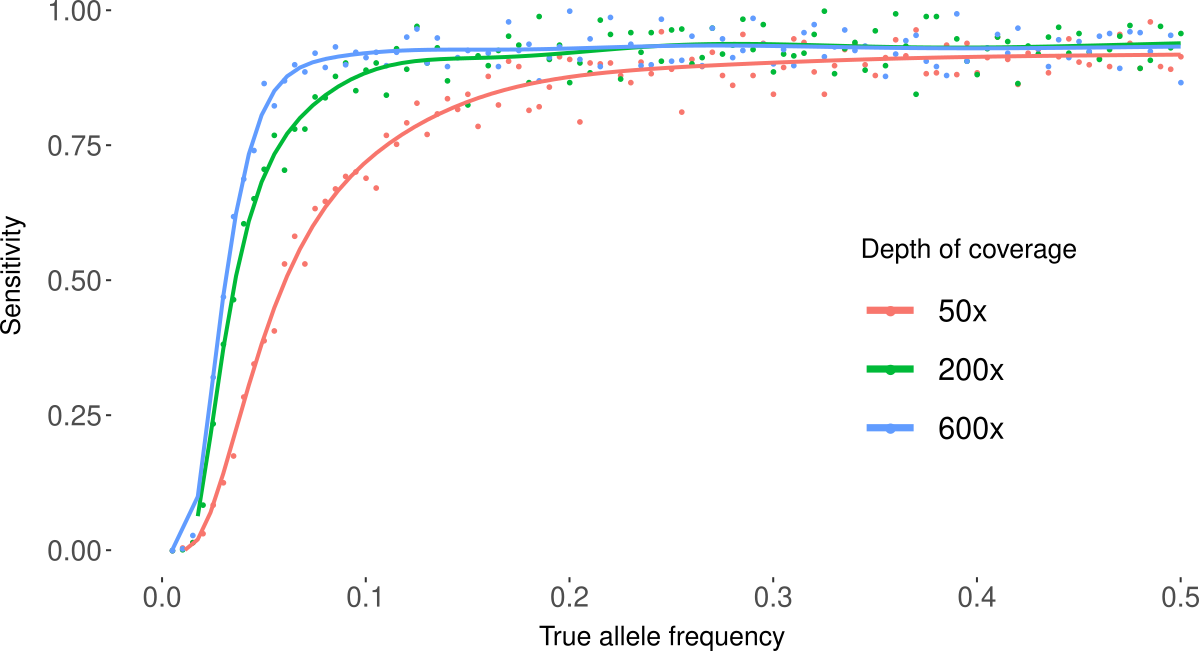

Supplement: zcad051_Supplemental_Files [file zcad051_supplemental_files.zip › sensitivityComparisonChr19_50x200x600x.png]

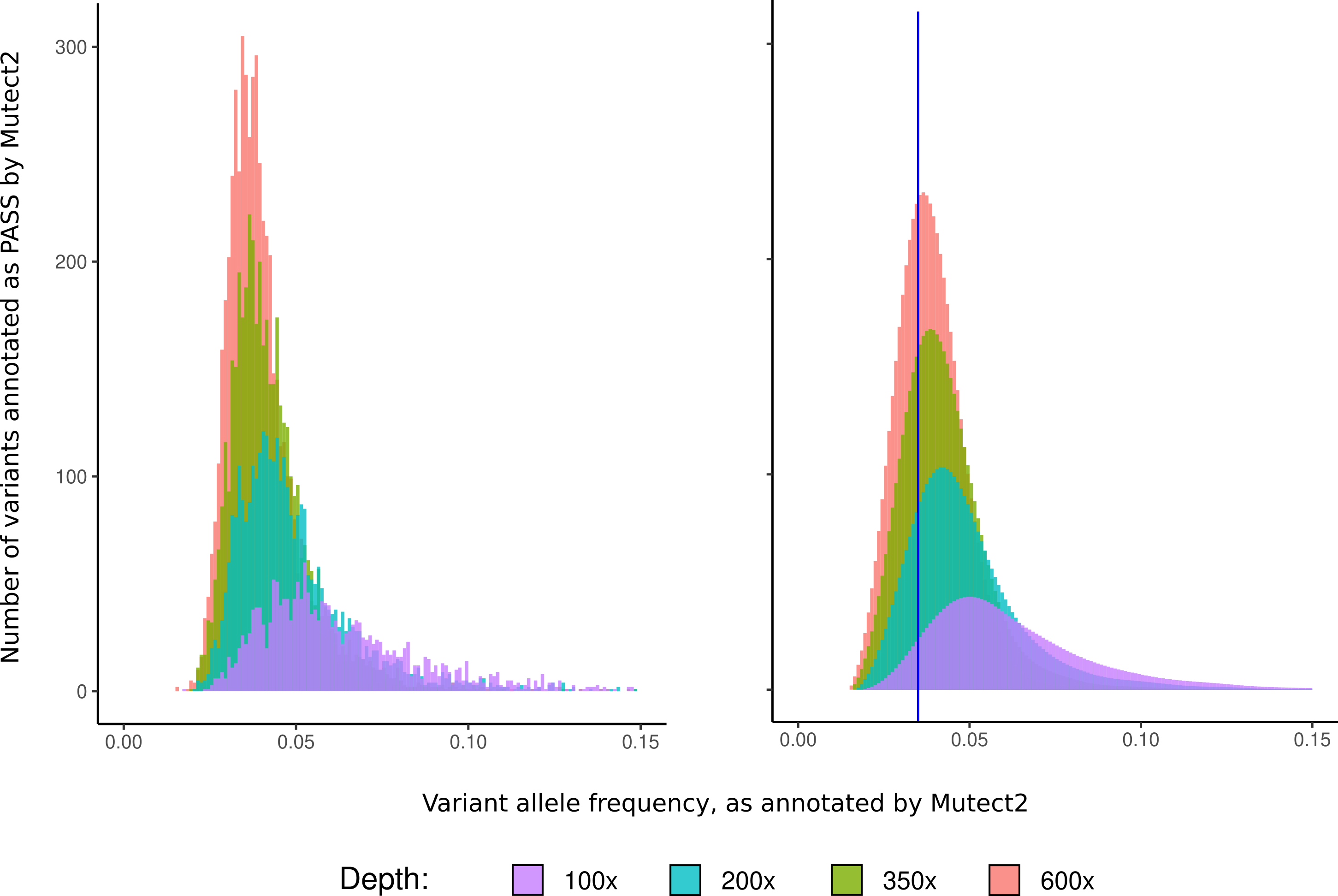

Supplement: zcad051_Supplemental_Files [file zcad051_supplemental_files.zip › suppFig1.png]
